# Predicting Packaging Material–Food Interactions and the Respective Migration and Permeation Based on Hansen Solubility Parameters—A Case Study of Bio-Based Polyester Cutin

**DOI:** 10.3390/polym17212961

**Published:** 2025-11-06

**Authors:** Costas Tsioptsias, Athanasios Goulas, Maria Tsini, Athanasia Zoglopiti, Anna Marinopoulou, Vassilis Karageorgiou

**Affiliations:** Department of Food Science and Technology, International Hellenic University, 57400 Sindos, Greece; agoulas@ihu.gr (A.G.); tsinimar@gmail.com (M.T.); amarinop@ihu.gr (A.M.); vkarageorgiou@ihu.gr (V.K.)

**Keywords:** compatibility, poly(lactic acid), poly(hydroxybutyrate), ester hydrolysis

## Abstract

One of the current and serious environmental problems is the pollution due to microplastics. There is an urgent need for biodegradable and bio-based materials for numerous applications, including food packaging. In this work we examine the bio-based polyester cutin for its potential to be used as food packaging material, in terms of migration, based on the Hansen Solubility Parameters (HSP). Cutin is a cross-linked polymer that is swelled by various solvents. We use the degree of swelling of cutin in carefully selected solvents of various polarities in order to estimate the HSP of cutin. Some solvents can induce alteration of the chemical structure of cutin, as proven by Fourier Transform Infrared (FTIR) measurements. This interferes with the process of estimation of the HSP and is discussed in depth. The distance R_a_ and the Relative Energy Difference (RED) between the HSP of cutin and various food components are calculated and used to predict the existence of favorable interactions between cutin and the food components, which is translated to a high probability for the existence of migration and permeation. Experimental confirmation of one prediction based on HSP is provided by UV-VIS photometry. Similar calculations were performed for other polyesters (poly(lactic acid) and poly(hydroxy butyrate)). Cutin exhibits compatibility with substances of low polarity, such as fats and lipids and non-polar compounds found in essential oils. Thus, migration into fatty foods is expected as well as sorption and permeation of some (volatile) compounds into cutin. Nevertheless, we conclude that the overall migration risk for cutin is lower than the one of other bio-based polyesters. HSP can be used for initial screening of potential migration risks; however, further research is necessary in order to assess the occurrence, extent, and significance of the actual migration.

## 1. Introduction

Petroleum-based polymeric materials offer various advantages over other materials, such as low cost, ease of processing, light weight, mechanical strength, etc. However, traditional synthetic polymeric materials are the root cause of the various environmental issues, e.g., the pollution from microplastics and the difficulty of recyclability (polymers, in order to be recycled, must fulfill some minimum requirements, and even in the case they do, the recycling is typically related to deteriorated properties). Thus, alternative renewable, natural, and bio-based polymers are needed so as to replace traditional plastics in numerous applications, including food packaging materials. Cutin is such a polymer.

Cutin is a cross-linked natural polyester found in plants, coating their leaves, fruits, flowers, and non-woody stems as a major structural constituent of their outer cell walls. This cuticular layer plays a central role in the terrestrial adaptation of plants by regulating aspiration and moisture transfer of their epidermal tissues [1,2]. Structurally, the plant cuticle is a hydrophobic composite material consisting of polysaccharides, waxes, polyesters, pectin, phenolic compounds, and flavonoids [3]. Among plant-derived biopolymers, cutin, as part of the plant cell walls, is an abundant biopolyester with a more complex and intricate chemical structure compared to other polymers such as cellulose or lignin. It is water-insoluble, composed of oxygenated long-chain epoxy- and hydroxy-fatty acids, commonly C16- or C18-, with a terminal hydroxyl group (ω-OH) and one or more unsaturated bonds [4,5]. Other monomers such as unsubstituted fatty acids, dicarboxylic acids, primary alcohols, and aldehyde derivatives of fatty acids are also present, further diversifying the polymer [1,6]. With concentrations ranging from ~0.5 to 1500 μg/cm^2^ of plant surface [1], cutin is widespread in nature, underscoring its ubiquity across agricultural crops. Observations of plant tissues using X-ray diffraction (XRD) and transmission electron microscopy (TEM) showed cutin to be amorphous, elastic, and reticulated, with these forms usually coexisting in layered arrangements, intermixed and interacting with the other structural components of the cell walls. Within these structures, cutin undergoes esterification, producing linear chains, with branching at intermediate hydroxyl groups and cross-linking between chains due to dicarboxylic acids. This creates a polymeric network that interacts with or entraps other components of the epidermis of plant cells [1,6].

Cutin can be extracted from various sources such as tomatoes (*Solanum lycopersicum*), peppers (*Capsicum annuum*), lime (*Citrus aurantifolia*), cherries (*Prunus avium*), lotus fruits (*Nelumbo nucifera*), and apples (*Malus pumila*) using a variety of methods described in the literature [3,7,8]. Due to the complexity of cuticular tissues, where cutin, waxes, and polysaccharides are intermixed into a dense matrix, the cutin isolation methodologies presented in the literature typically involve dewaxing with organic solvents and acidic polysaccharide hydrolysis prior to cutin depolymerization using a variety of approaches, such as aqueous alkaline hydrolysis, methanol transesterification with BF_3_ or NaOCH_3_, reductive cleavage by treatment with LiAlH_4_ in tetrahydrofuran, or trimethylsilyl iodide in organic solvents [3]. Among the proposed methodologies, the one employed in the presented study, described in the following section, was chosen based on its simplicity and potential applicability on a large scale.

The development of biodegradable packaging materials, as a partial replacement for petroleum-based synthetic polymers, presents a significant challenge. Given the pressing environmental need to promote a circular economy and sustainability, it is essential to design innovative materials derived from renewable natural resources such as starch, collagen, chitosan, proteins, polysaccharides, aliphatic polyesters, and others. However, natural raw materials are not inherently structured for direct use and therefore require appropriate pretreatment to render them processable and suitable for the fabrication of new materials—and consequently, new packaging products. Similarly, synthetic polymers also require modification during their formulation with the incorporation of various additives—such as antioxidants, anti-aging agents, plasticizers, structural stabilizers, surface modifiers, and pigments—to tailor their properties for specific applications. Many of these additives have been associated with potential adverse effects on human health following prolonged exposure. Moreover, any material intended for packaging applications, particularly within the food industry, must comply with strict requirements regarding its physicochemical characteristics—including mechanical, thermal, and barrier properties—as well as its potential interactions with the packaged food. Cutin is a non-toxic, biodegradable, insoluble, amorphous substance that provides UV protection and is regarded as a promising alternative to conventional plastics for certain applications [9]. Cutin, on its own or combined with other ingredients, mainly biopolymers, such as pectin, chitin/chitosan, and zein, but also with smaller-sized constituents such as glycerol, has been investigated for the development of biodegradable materials either in the form of self-standing membranes or as lacquers of glass or metal containers [8,10,11,12,13,14]. In most cases, for the preparation of cutin-based materials, the hydrolyzed cutin, as extracted from the plant cell walls, undergoes a heat condensation polymerization process, which, when completed, results in a polyester material with good flexibility, hydrophobicity, mechanical stability, and controlled permeability characteristics.

The Hansen Solubility Parameters (HSPs) are widely used in academia and industry for finding suitable solvents for various applications. The total HSP is calculated from three partial HSPs, one accounting for dispersion forces, one for polar interactions, and one for hydrogen bonding [15]. The concept of HSPs is rather simple and is based on the basic rule of chemistry that “like dissolves like”. Substances with similar HSPs are likely to exhibit mutual solubility and compatibility. HSPs have been used for numerous applications, e.g., for finding solvents for extraction purposes, including analytical purposes [16,17,18]; finding solvents for depolymerization of lignocellulosic biomass [19]; and finding good solvents for polymers [20], paints, pharmaceuticals, and other [15]. Solvent screening is perhaps the most traditional application of HSP. However, HSP actually predicts the compatibility/miscibility of two substances and thus indirectly can predict the existence of favorable interactions between two substances. Thus, besides solvent screening, HSPs have been used for other applications, e.g., permeation, including skin permeation, composite materials, polymer adhesion, and others [15,21].

Despite their broad range of applications, the use of HSP in the field of packaging is rather limited. HSPs have been used for finding suitable solvents for the recycling of food packaging containers [22,23]. Also, HSPs, along with molecular dynamics simulations and artificial neural networks, have been used to study migration in food simulants [24]. HSPs have also been used to study the permeation of migrants through polymeric films [25]. In the webpage for the HSP, the use of HSP for studying flavor losses is discussed [21].

In order for a material to be used in food packaging, it must exhibit certain properties, e.g., suitable mechanical strength, low permeability, etc. Ideally, the material should be inert for the food; that is, there should not exist any interaction between the packaging material and the food in order to avoid problems of migration from the packaging to the food and problems of sorption and permeation from the food out of the packaging. Favorable interactions between the potential migrants and food suggest compatibility, and consequently, a high solubility and a high tendency for solubility of the migrant into food are expected. On the contrary, in the case of poor interactions between migrants and food, a low risk of migration is expected. Similarly, if some of the food’s volatile compounds exhibit favorable interactions and compatibility with the packaging polymeric material, then there is a high probability that they will be adsorbed/absorbed in the polymer. This (the trapping of food compounds into packaging material) can cause considerable flavor losses. In the webpage of HSP [21], a real case of considerable flavor losses in a natural juice is discussed in detail. In addition, the adsorption of food compounds into the packaging material is the first stage for the occurrence of permeation. Adsorption occurs for enthalpic reasons while diffusion is an entropy-driven process. Thus, small molecules, due to the driving force of entropy, will diffuse from the surface into the bulk of the material and finally permeate it.

Migration risk can be evaluated both experimentally and theoretically. In the 10/2011 European regulation about plastic materials in contact with foods, six food simulants are proposed to study migration. The material is immersed in the food simulant (mixtures of water and ethanol or acetic acid for hydrophilic foods and vegetable oils and an aqueous mixture of 50% *v*/*v* ethanol for lipophilic foods), and the migration is assessed by the mass loss. This concerns the overall migration evaluation. Evaluation of the specific migration of some substance or group of similar substances is performed by appropriate analytical procedures (e.g., photometry, chromatography, etc.). Theoretically, the study of migration is typically performed by models based on diffusion [26,27]. This requires the knowledge of the diffusion coefficient of various substances; however, such reliable data are not always available. As mentioned above, there is a limited number of studies where HSPs have been used to predict or study migration or permeation effects [21,24,25]. In addition, these methodologies are not solely based on HSP but were used in combination with other theoretical approaches, e.g., molecular dynamics. Such approaches, as well as the diffusion-based models, are complex and require a rather strong background in Physical Chemistry, Transport Phenomena, etc., and thus can be used only by experts. In addition, in such sophisticated modeling, typically, various assumptions are needed; thus, the predictive ability of such approaches heavily depends on parameter fitting on related available experimental data. However, accurate data for the diffusion of substances into foods or of food components into polymers is rather limited. Also, such models are very useful for studying specific cases, but any general applicability should be handled with care. On the contrary, the prediction of substance compatibility with HSP is a very simple and rapid procedure that can be used by non-experts in Physical Chemistry, etc. Also, for various polymer materials and common food components (fats, carbohydrates, etc.), the values of HSPs have been derived from actual thermodynamic (solubility or swelling) data. Thus, despite their simplicity, HSPs exhibit a remarkable predictive ability.

HSPs could be very effective for a general applicability initial screening of migration and permeation risks. For example, if the HSP of the polymeric packaging material is very close to the HSP of some group components, e.g., fats, this suggests that it would not be a good idea to package fatty foods in this material. Thus, the scope of this work is to propose a simple methodology of general applicability for initial screening of migration/permeation risks in polymeric materials. The methodology is simply based on considering various groups of food components and using their average values of HSP for comparison with the HSP of the candidate packaging material. The methodology is applied to explore the potential of the bio-based polymer cutin as food packaging in terms of migration/permeation. For this purpose, the HSP of cutin is firstly determined from swelling data by various solvents. Then, these are compared with the HSP of numerous food components in order to predict with which substances favorable interactions (and thus migration) are likely to exist. Experimental confirmation for one of the predictions, as well as comparison with other bio-based polyesters, is also provided.

## 2. Materials and Methods

### 2.1. Materials

Diethyl ether, hexane, ethyl acetate, isopropanol, acetone, and chloroform were all of analytical grade purity purchased either from Carlo Erba Reagenti (Italy), Fisher Scientific (Germany), or Sigma-Aldrich Chemie GmbH (Germany).

### 2.2. Cutin Extraction and Preparation of Films

The cutin used in the present study was extracted from pepper peels, the outer skin of the pepper fruit, using non-marketable, substandard mixed-type peppers with the following methodology. Non-marketable peppers were immersed in a 2% *w*/*w* citric acid solution and heated in a static vertical autoclave (Korimat, Mittenaar, Germany) at 125 °C for 2 h. The liquefied flesh of the peppers was then separated from the peels, stems, and seeds using a refiner equipped with 0.5 mm pore diameter sieves (Henri Biaugeaud, France). Subsequently, the collected peels were washed with potable water to remove flesh residues and separated/collected free from stems and seeds by gravitational separation in a water tank. The clean peels were then dried in a tray dryer (APEX Construction Ltd., Uxbridge, UK) at 25–30 °C before any further processing.

Cutin was isolated from the dried peels with alkaline hydrolysis according to the method described in the literature [6,7] with some modifications. Briefly, the dehydrated pepper skins were impregnated in an aqueous sodium hydroxide solution (NaOH, 3% *w*/*w*), at a peel-to-solution weight ratio of ≈1:10, and heated at 125 °C for 2 h. The solid peel residue was then removed by filtration and discarded, while the filtrate was further clarified from the suspended solids by centrifugation (Sorvall RC-7, Thermo Scientific, Waltham, MA, USA) at 4000× *g*. The hydrolyzed cutin, present in the alkaline supernatant solution, was then precipitated and separated as solid residue after neutralization and slight acidification of the solution between pH 5 and 4.5, with concentrated hydrochloric acid (33% *w*/*w*). The precipitate was then recovered by centrifugation at 4500× *g* and washed from salts and other water-soluble components with distilled water. Crude cutin was then freeze-dried (Gamma 1–20, Martin Christ, Osterode am Harz, Germany) prior to any further processing for the purposes of the present work. For the crude pepper cutin, ^1^H-NMR spectra were recorded on an Agilent 500 spectrometer (DD2) with ^1^H at 500 MHz and ^13^C at 126 MHz, using the TMS internal standard and deuterated chloroform as the solvent at 13 °C.

Cutin film preparation was performed via a heat condensation polymerization process at 150 °C, in polytetrafluoroethylene (PTFE) containers, where 0.093 g of cutin/cm^2^ of surface was weighted and heated in an oven (Gallenkamp, model OV-160, Cambridge, Cambridgeshire, UK) for a period of 6 h. The monomer of cutin is a dihydroxy acid with the OH groups at positions 10 and 16 (which is the end group). The polymerization reaction is based on the esterification reaction between COOH at position 1 with the two OH groups. This leads to the formation of a cross-linked polyester. A scheme for the polymerization reaction is presented in Figure S1 of the Supplementary Materials.

### 2.3. Swelling Experiments

Pieces of cutin with dimensions of around 1 cm length, 0.6 cm width, and 0.1 cm thickness were immersed in excess (~10 mL) of seven different solvents (hexane, diethyl ether, chloroform, acetone, ethyl acetate, isopropanol, and water) for 24 h at room temperature. The exact dimensions of the samples before and after, the immersion were measured with a caliper (±0.01 mm). The volume of the samples was calculated by the dimensions. The degree of swelling (% Swelling) in each solvent was calculated from the following equation:

(1) $$\%\;Swelling=\frac{V_{after}-V_{before}}{V_{before}}$$

where

$V_{before}$ is the volume of the dry sample before the immersion;

$V_{after}$ is the volume of the wet sample after the immersion.

The swelling experiments were performed in two independent replicate experiments.

### 2.4. Spectroscopic Measurements

In order to examine the interactions of cutin with water, small pieces of cutin membranes (after polymerization) were immersed in excess of water of three different pH values: (a) one sample was immersed in water of pH = 1 adjusted by the addition of concentrated HCl acid solution, (b) one sample was immersed in water of pH = 13 adjusted by the addition of NaOH and (c) one sample was immersed in distilled water without any pH adjustment. After immersion for 24 h at room temperature, these samples were air-dried and along with a non-immersed cutin membrane sample, were examined by a Nicolet 380 Attenuated Total Reflectance Fourier Transform Infrared (ATR-FTIR, Waltham, MA, USA) spectrometer with a resolution of 4 cm^−1^ and 32 scans.

Finally, in order to examine possible interaction of cutin with fats, a piece of cutin film was immersed in sunflower oil (food grade purchased from the local market) and kept airtight for a 6-month period at room temperature. A reference sample (pure sunflower oil) was also kept sealed under identical conditions. After six months the pure sunflower oil was used as the blank sample for measuring the light absorbance of the sunflower oil that was in contact with the cutin membrane in a HACH DR 3900 UV-VIS spectrophotometer (Loveland, CO, USA). The absorbance of the sample was measured at 400, 550, and 700 nm in order to examine wavelengths over the entire range of the visible spectrum.

### 2.5. Estimation of the HSP of Cutin

Cutin is a cross-linked polymer and as such, it is not dissolved, but it can be swelled by good solvents. HSPs, besides solubility data, can be estimated from swelling data [15]. The R_a_ distance (same units as the HSP) between cutin and a good solvent will be low while the R_a_ value between cutin and a bad solvent will be higher. The R_a_ distance is calculated by the following equation [15]:

(2) $$R_{a}=\sqrt{4{\left(\delta_{d1}-\delta_{d2}\right)}^{2}+{\left(\delta_{p1}-\delta_{p2}\right)}^{2}+{\left(\delta_{hb1}-\delta_{hb2}\right)}^{2}}$$

where

$\delta_{d}$ is the dispersion Hansen Solubility Parameter (of the substance 1 and 2, respectively, according to the subscript) in MPa^1/2^.

$\delta_{p}$ is the polar Hansen Solubility Parameter in MPa^1/2^.

$\delta_{hb}$ is the hydrogen bonding Hansen Solubility Parameter in MPa^1/2^.

Thus, we use the following concept in order to estimate the HSP of cutin: the % degree of swelling and the R_a_ should be negatively correlated. The subscript 1 in Equation (2) refers to cutin, and the subscript 2 refers to the solvent. Initially the values of the three HSP of cutin are set arbitrarily equal to 5. Then, the six values of R_a_ distance are calculated for the six solvents [15] (for reasons discussed in detail in Section 3.1, water was not included). The R_a_ values are plotted against the % degree of swelling, and then linear regression is performed and the R^2^ value is calculated. Then the values of HSP of cutin are altered, and by trial and error the values of HSP that lead to the maximization of R^2^ are obtained. In order to facilitate the optimization procedure, we used the constraint that the values of HSP of cutin should be in the range of 2.5 to 40 (this is the range of the typical values of HSP for the vast majority of substances). For reasons that will be discussed in detail in Section 3.1, the above procedure was performed two times in total: one time with six solvents (water was excluded) and one time with five solvents (water and isopropanol were excluded). Thus, two different sets of the HSP of cutin were estimated.

### 2.6. Predicting Migration with the HSP

As mentioned above, lower values of $R_{a}$ suggest similarity of substance 1 and 2 and thus the lower the $R_{a}$ value is, the more likely compatibility/miscibility between the two substances is to exist. Here, the following question arises: How low should the R_a_ value be in order for compatibility to exist? This question has no definite answer, and for this reason, the concept of Relative Energy Difference (RED) is quite useful. Specifically, the R_a_ value can be compared with a reference distance value (often determined based on experimental data) to calculate the Relative Energy Difference (RED) by the following equation [15]:

(3) $$RED=\frac{R_{a}}{R_{o}}$$

where

R_o_: Reference distance value (Radius of interaction sphere in Hansen space).

RED values lower than 1 suggest high affinity for the substances involved in the calculation of R_a_, while RED values higher than 1 indicate poor compatibility (the higher the RED value is, the lower the compatibility). For reasons explained in Section 3.1, the distance R_a_ of the HSP of cutin and hexane was used as the R_o_. Forty-seven (47) food components were categorized in six groups: carbohydrates, fats and lipids, amino acids, vitamins, polar essential oil components, and non-polar essential oil components. For each substance, the R_a_ value was calculated. Then an average R_a_ value was calculated for each group of substances. Then the RED value for each group was calculated (as mentioned above using the R_a_ value of cutin-hexane as the R_o_ value). By comparing the RED value with 1, it can be predicted with which group of substances cutin is likely to exhibit favorable interactions and compatibility. The existence of strong interactions suggests a high probability of migration and/or permeation.

## 3. Results and Discussion

### 3.1. Estimation of HSP of Cutin

**Figure 1:**
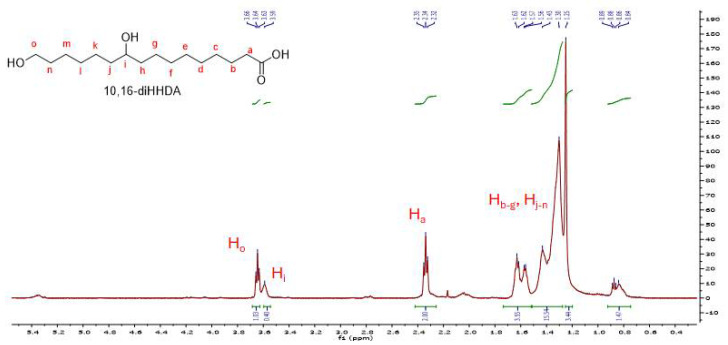
^1^H NMR spectra of the 10,16-dihydroxy hexadecanoic acid (10,16-diHHDA) from the cutin extract in chloroform.

The NMR spectrum of cutin extract in chloroform is presented in Figure 1. Chemical shifts are given in δ values (ppm) referenced to CDCl_3_ with 7.26 for ^1^H and 77.10 for ^13^C. Coupling constants (*J*) are reported in Hz. The observed chemical shifts (indicated in Figure 1) are in good agreement with spectra previously reported in the literature [28] for cutin extracted from tomato. The observed structure most closely corresponds to the observed data being that of the 10,16-dihydroxy hexadecanoic acid (10,16-diHHDA).

**Figure 2:**
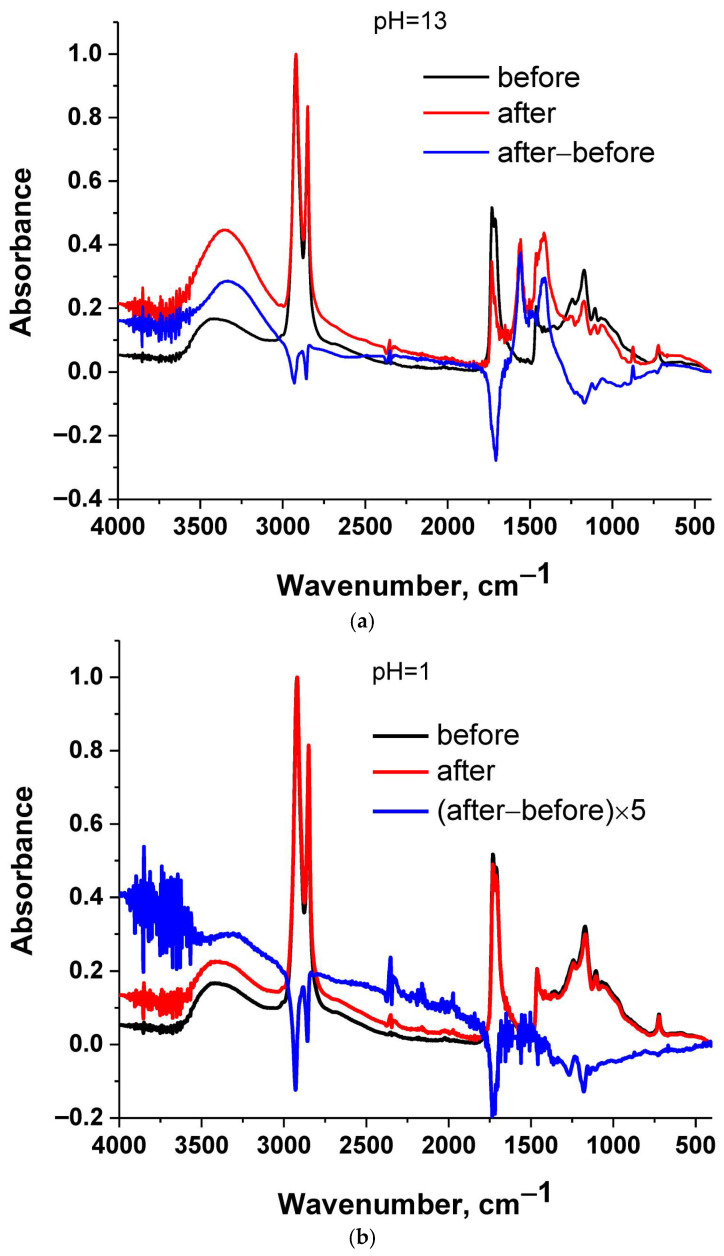

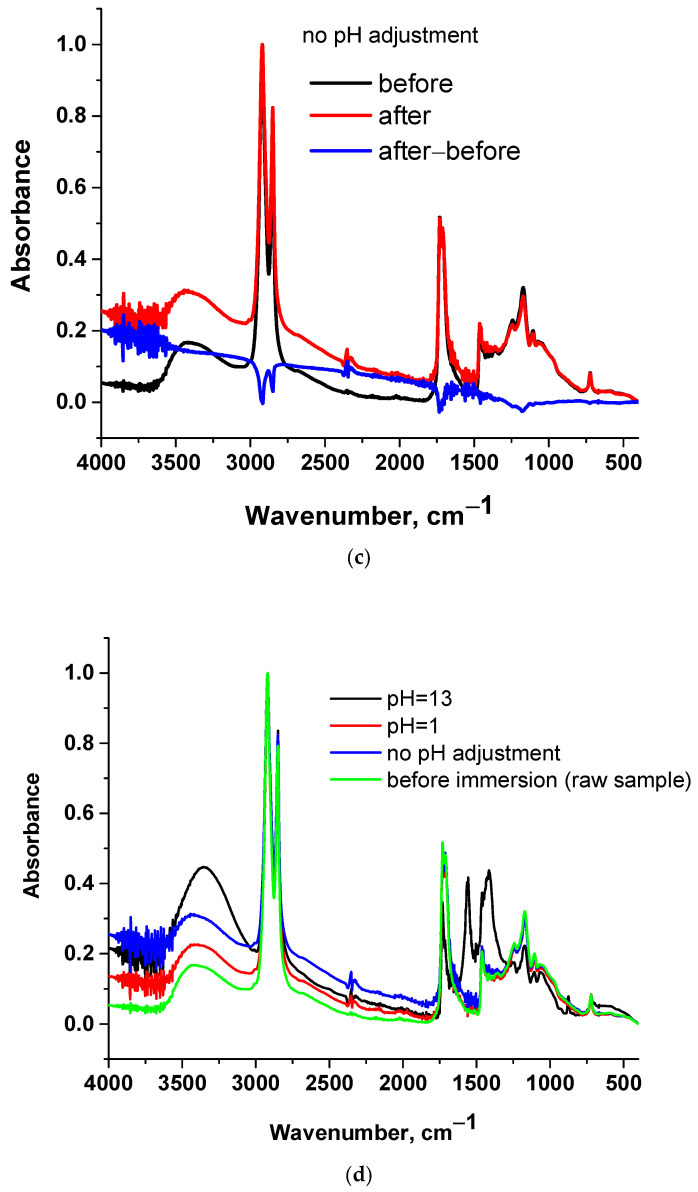
FTIR spectra of cutin before and after immersion in water and their subtracted spectrum: (**a**) pH = 13, (**b**) pH = 1, (**c**) no pH adjustment and (**d**) all samples.

In Figure 2 the FTIR spectrum of cutin is presented. A broad peak at around 3300 cm^−1^ can be observed, which is assigned to O-H stretching in alcohols and acids and water. The band around 2900 cm^−1^ is assigned to C-H stretching, while the band at 1730 cm^−1^ is typical of ester C=O stretching [29]. At around 1400 cm^−1^, C-H vibrations occur, and in the region 1000–1200 cm^−1^, the C-O stretching vibration of alcohols, acids, and esters occurs. The presence of these groups is in agreement with the NMR data.

Cutin is a polyester. In general, polyesters are susceptible to hydrolysis. If cutin is hydrolyzed, its chemical structure is altered during the swelling experiment; thus, the swelling degree in water and in the other solvents will not refer to the exact same structure. In addition, HSPs have been developed for predicting physical interactions and not chemical reactivity, and thus water may not be correct to be included for the estimation of the HSP of cutin. Thus, it was checked by FTIR if cutin is hydrolyzed during swelling by water.

In Figure 2 the FTIR spectra of cutin before and after immersion in water of various pH values for 24 h are presented. Along with the raw spectra, the subtracted spectrum is presented, which facilitates tracking the differences between the samples before and after the immersion. Spectra subtraction is a suitable method for detecting differences between samples [29]. As can be seen in the subtracted spectra (Figure 2a), at pH = 13 a negative peak is present at around 1730 cm^−1^ which is assigned to ester C=O stretching [29]. A positive broad peak at around 3400 cm^−1^ is also present, which is assigned to O-H stretching [29]. These suggest a decrease in ester bonds and an increase in OH groups. Both of these can be explained in terms of (partial) hydrolysis of cutin. For other pH values (Figure 2b,c) similar conclusions can be extracted, but the differences are smaller; e.g., for pH = 1 the differences are more evident if the subtracted spectrum is multiplied by a factor of 5. These are in agreement with optical observations of the water after the immersion. Specifically, the water with pH = 13 exhibited a slight yellow-brown color, which can be explained in terms of a higher degree of hydrolysis and thus higher migration and solubilization in water of some low molecular weight fragments. Thus, it seems that at higher pH values, hydrolysis is more extensive (in accordance also with the methods described for cutin extraction), but it occurs to a smaller extent at lower pH values as well. In Figure 2d, all four spectra are presented. It can be seen that only in the case of the sample immersed in water of pH = 13 can considerable changes be observed. All other spectra seem similar; however, as discussed above, by spectrum subtraction, some minor changes can be detected.

**Figure 3:**
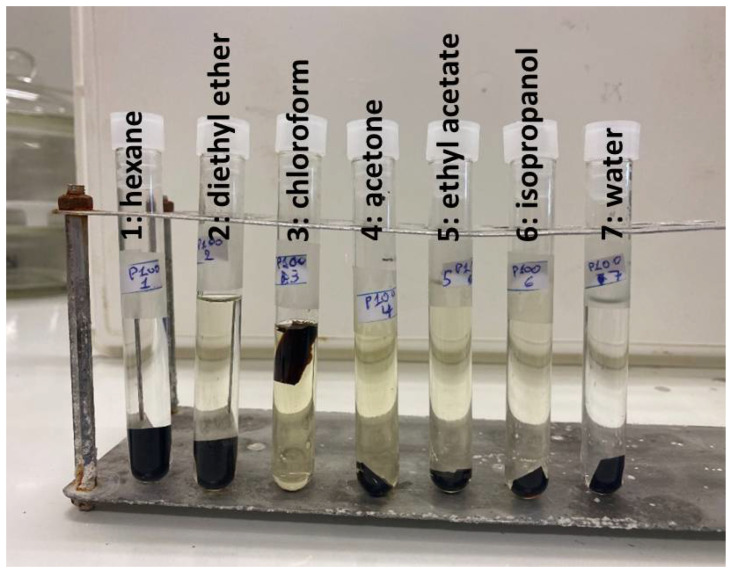
Samples of cutin after being immersed in solvents. From left to right the solvents are 1: hexane, 2: diethyl ether, 3: chloroform, 4: acetone, 5: ethyl acetate, 6: isopropanol and 7: water.

Since water alters the chemical structure of cutin during swelling, as mentioned above, the % swelling in water and the % swelling in the other solvents will not correspond to the exact same structure. Thus, although the % degree of swelling of cutin membranes immersed in water was minimal (≈10%), it was decided to exclude water from the calculations. In Table 1, the % swelling of cutin films in various solvents is presented. Photos of the swollen samples inside the solvents are presented in Figure 3. As can be seen, chloroform appears to be the best solvent since it causes by far the highest swelling (Table 1). Also, as can be seen in Figure 3, chloroform (solvent 3) exhibited the darker color at the end of the swelling experiments. Other low-medium polarity solvents like diethyl ether, acetone and ethyl acetate can adequately swell cutin to an extent in the range of 50–60%. The nonpolar solvent hexane causes a minimum swelling of 10–20%, suggesting that hexane is a poor solvent for cutin and weak interactions exist between the two. For this reason, the R_a_ of hexane was used as the R_o_ value for the calculation of RED as mentioned in Section 3.2. In other words, if the RED value of a substance is higher than 1, this means that the interactions with cutin will be even poorer than the ones of hexane. Thus, very poor interactions would be expected with cutin and thus minimum migration.

**Figure 4:**
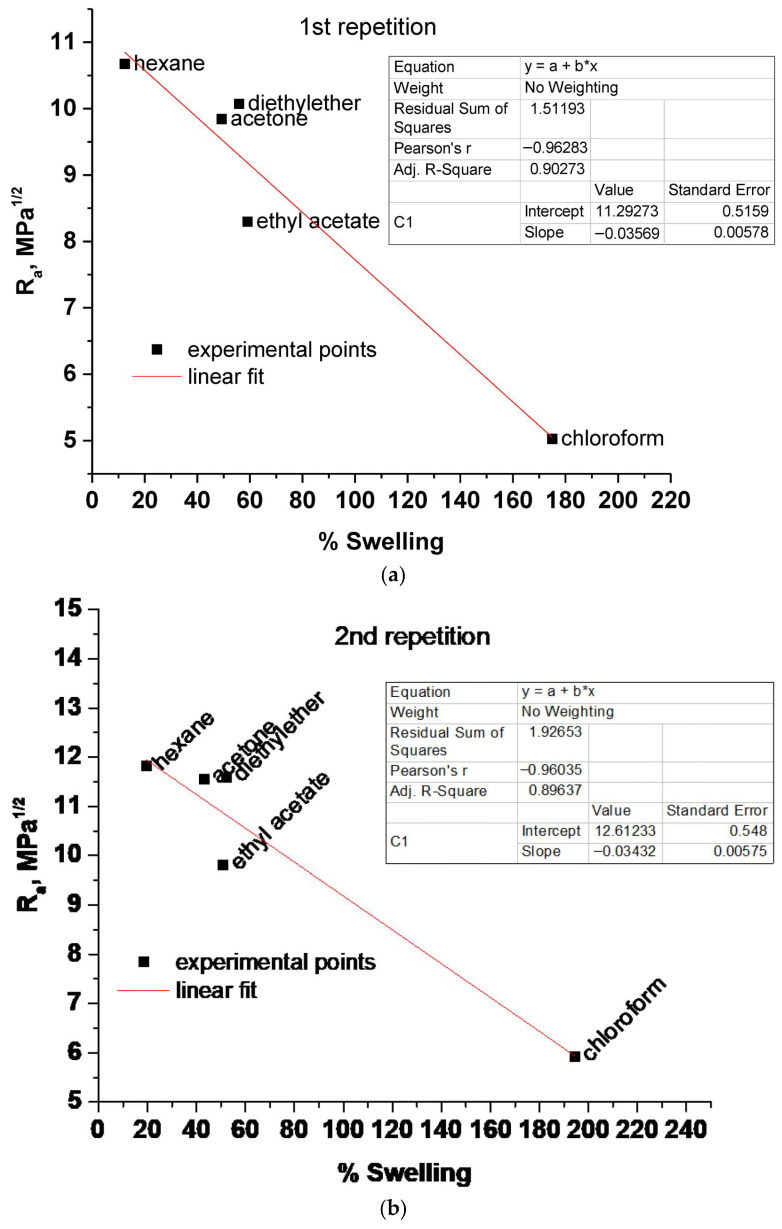
Correlation plot of R_a_ and % swelling of cutin in five different solvents: (**a**) 1st repetition and (**b**) 2nd repetition.

Interestingly, isopropanol appears to be the second-best solvent after chloroform. However, if chloroform is the best solvent, it would be expected that the second-best solvent would also be a medium polarity solvent like ethyl acetate and not a rather high polarity solvent like isopropanol. In addition, the color of isopropanol after the immersion and swelling is not as intense as for other solvents. These comparisons created suspicions that the % swelling in isopropanol may not be representative. An explanation for this would be a similar explanation as for water. Polyesters, besides hydrolysis of ester bonds, are also susceptible to transesterification reactions. Thus, it seems probable that isopropanol, like water, also affects the chemical structure of cutin, and thus the % swelling in isopropanol and in other solvents does not refer to the exact same structure. In any case, as mentioned in Section 3.1, two sets of HSP of cutin were estimated from the swelling data of six solvents (excluding only water) and from five solvents (excluding water and isopropanol). As can be seen, for the case of five solvents, in Figure 4, the correlation plots of R_a_ and % swelling are presented for the two swelling repetition experiments.

By the procedure described in Section 3.1 two sets of HSP of cutin were estimated and are presented in Table 2.

As can be seen, there is a fairly good agreement between the values of HSP calculated from the two repetition swelling experiments (for each set of values). In the case of 6 solvents (propanol included), there is a deviation between the δ_d_ values. In any case, reasonable values were estimated from both five and six solvents. Compared to other bio-based and biodegradable polyesters like poly(lactic acid) (PLA), poly(hydroxy butyrate) (PHB), and poly(ε-caprolactone) (PCL), the values of δ_d_ estimated from the five solvents are closer. For example, a PLA sample exhibited values of δ_d_, δ_p_ and δ_hb_ respectively equal to 17.9, 9.2, and 5.9 [30]. Also, various other polyesters have been reported [30] to have similar δ_d_ values and a δ_p_ higher than δ_hb_ as the ones calculated for cutin from five solvents. On the contrary, the HSP calculated from the six solvents yielded values of δ_hb_ quite higher than the δ_p_. This most likely arises from the high degree of swelling in isopropanol and the high δ_hb_ of isopropanol that influences the regression procedure. Thus, it seems that the set of HSP calculated from the five solvents is more reliable. Finally, it is worth mentioning that the lower values of δ_p_ and δ_hb_ of cutin compared to other polyesters most likely arise from the cross-linked structure of cutin, since due to cross-linking, fewer free groups are available for taking part in intermolecular interactions such as polar interactions and hydrogen bonding.

### 3.2. Predicting Migration and Permeation Based on HSP

From the estimated values of the HSP of cutin (from the five solvents) and the available HSP of various food components, the R_a_ distance was calculated. The food components were categorized in six groups. For each group, an average R_a_ value was also calculated. These R_a_ values are presented in Table 3. In the majority of cases the average R_a_ value is representative for most of the substances of the group.

**Figure 5:**
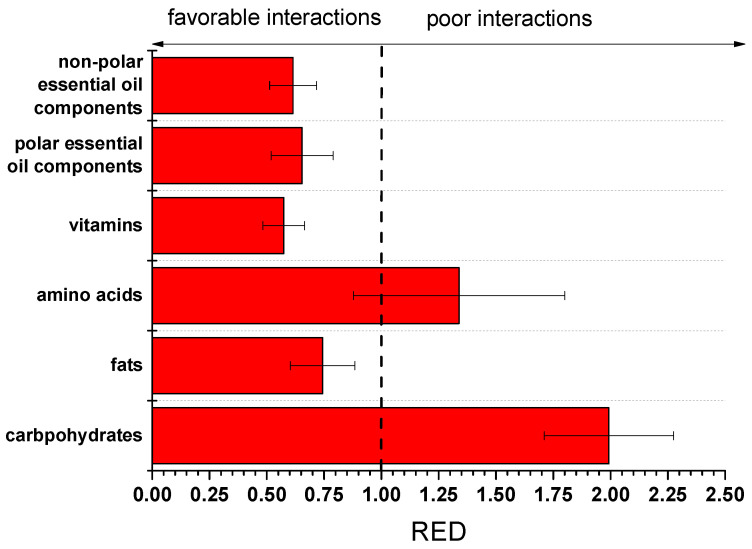
RED values of various groups of food components with cutin.

In order to facilitate the evaluation of the data of Table 3, the RED values were calculated for the average values of HSP of cutin (from the five solvents) and the average values of the groups (and by using the R_a_ of hexane and cutin as the R_o_ value). The RED values are presented in Figure 5.

It is recalled that RED values higher than 1 suggest poor compatibility, while values lower than 1 suggest good compatibility (the lower the value is, the better the compatibility). As can be seen, cutin exhibits RED values lower than 1 with various groups of food components such as fats, vitamins, and both polar and non-polar essential oil components. Carbohydrates and amino acids (both are highly polar) seem to interact to a lesser extent with cutin (except, of course, if chemical reactions take place, e.g., transesterification). However, the uncertainty of the RED value for these groups is partially lower than 1; thus, interaction may exist with some of the components of these groups.

In order to provide some confirmation for one of the predictions, a piece of cutin was immersed in sunflower oil for a 6-month period at room temperature, since, based on the predictions, cutin will strongly interact with fat and lipids, and thus migration in such oily foods would be expected. A reference sunflower oil without being in contact with cutin was also stored under identical conditions. This reference sample was used as a blank sample in the UV-VIS photometer, and the absorbance at three different wavelengths (400, 550, and 700 nm) covering the entire range of visible light was measured. In the oil sample, which was in contact with cutin, a minor increase in its color was hardly detectable by the naked eye; however, based on the UV-VIS measurements, which are presented in Table 4, it is clear that the color increased. This clearly suggests migration from cutin to the oil.

## 4. Further Discussion

Based on the FTIR results and discussion of Section 3.1, it appears that cutin can be affected by water. Esterification/hydrolysis is a two-way reaction that should reach equilibrium and not continue over time. The degree of hydrolysis should be quantified and it must be checked whether this hydrolysis affects other properties, e.g., mechanical, or if it is practically negligible. These will allow us to evaluate if cutin can be used for high water content foods.

**Figure 6:**
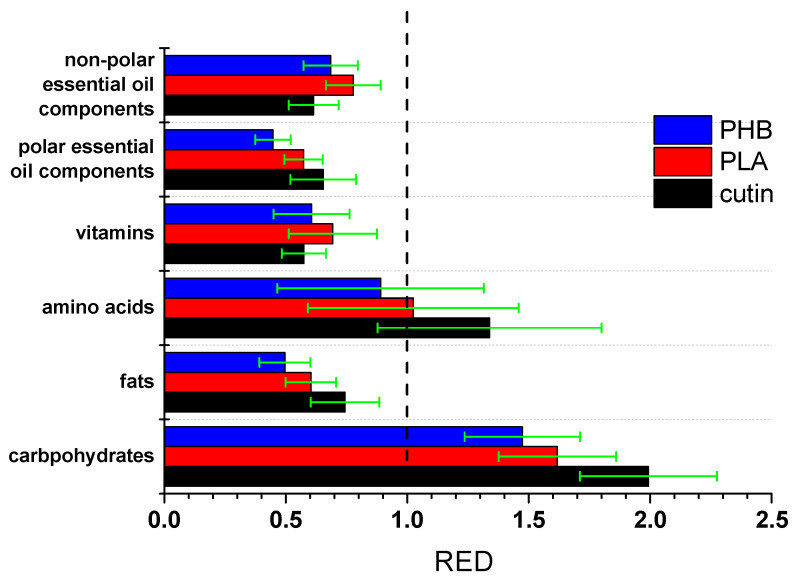
RED values of various groups of food components with cutin, PLA, and PHB.

It should be stressed that HSPs are quite useful; however, obviously, they are not always successful, and the corresponding predictions cannot be taken for granted. In any case, based on the HSP predictions, cutin seems to have good compatibility with polar and non-polar essential oil components, various vitamins, fats, and lipids. Thus, both migration of cutin into the food and adsorption of such molecules on the surface of the cutin material are potentially possible, and for the case of lipids, this was experimentally confirmed. Also, high permeation would be expected for foods rich in non-polar essential oil compounds. Of course, such interactions and migration should be further studied and quantified. Another major aspect, e.g., for the case of migration to sunflower oil, is whether this migration is continuous over time or it proceeds to some extent, reaches equilibrium, and stops. Furthermore, other aspects should also be taken into account. Specifically, in the ideal case of a fully cross-linked polymer with no impurities, a zero migration would be expected. A portion of the observed migration may be due to impurities or low molecular weight fragments of cutin. Optimization of the polymerization procedure so as to increase the degree of crosslinking of cutin and its molecular weight could assist in minimizing the migration risk. Also, it is worth mentioning that, if similar calculations are performed for other polyesters, e.g., PLA, similar conclusions or even stronger interactions would be obtained, e.g., PLA exhibits good compatibility with fats, vitamins, etc. The fact that cutin is cross-linked and in combination with the lower values of δ_p_ and δ_hb_ compared to other polyesters suggests that among such bio-based polyesters, cutin is expected to exhibit one of the lowest migration potentials. Indeed, the RED values for the same food components as above with PLA and poly(hydroxy butyrate) (PHB) were calculated from the reported values of HSP and interaction radius (R_o_) of these polymers [30]. The results are presented in Figure 6.

As can be seen, cutin exhibits lower average RED values (better compatibility) with non-polar essential oil components and marginally lower with vitamins. This is rather expected due to the low value of cutin’s δ_p_. However, for all other food component categories the average RED values of cutin are higher than the ones of the other polyesters. The error bars are rather high and cause a partial overlapping in some cases. These error bars arise from the standard deviation of the HSP of each group. Nevertheless, the average values clearly suggest a trend. Thus, it seems that cutin is less susceptible to migration than other bio-based polyesters.

Finally, it should be stressed that most food packing materials are composite multi-layered laminate materials, and thus migration issues could be surpassed by using cutin as a base and making a surface film from another polymer in order to avoid hydrolysis permeation etc.

## 5. Conclusions

Cutin is hydrolyzed by water to various extents depending on the pH value. Hydrolysis is more pronounced at high pH values. Thus, upon swelling by water, which appears to be minimal, the chemical structure of cutin is altered, and the % swelling in water and the % swelling in other solvents do not correspond to the same structure. According to that, water was excluded as a solvent from the estimation of HSPs of cutin, which were estimated based on the swelling data of various solvents. Similar problems may be present in the case of isopropanol due to transesterification reaction. The estimated HSPs of cutin, which were calculated by excluding water and isopropanol are more reasonable and closer to the ones of other polyesters. Based on the HSP and the RED values, it seems that cutin exhibits good compatibility with a variety of food components such as fats and lipids, vitamins, and polar and non-polar essential oil components. Experimental confirmation was provided for the case of lipids. Thus, migration from cutin to food, or adsorption and possibly permeation of (volatile) food components out of the packaging material, is potentially possible. Quantification of the degree of hydrolysis of cutin and its influence on other properties must be further investigated. Also, quantification of the migration to lipids should be carried out, and the other predictions should also be experimentally confirmed and studied, e.g., if migration stops or continues over time. Other bio-based polyesters exhibit even higher interactions with such components, partially due to their non-cross-linked structure and higher polar and hydrogen bond HSP. Thus, cutin is a very promising polyester with a lower migration risk than other widely used polyesters such as PLA.

## Figures and Tables

**Table 1:** % Swelling of cutin films in various solvents.

		% Swelling		
Number	Solvent	1st Repetition	2nd Repetition	Mean ± Standard Deviation
1	hexane	12	20	16 ± 5.7
2	diethylether	56	52	54 ± 2.8
3	chloroform	175	194	184.5 ± 13.4
4	acetone	49	43	46 ± 4.2
5	ethyl acetate	59	51	55 ± 6
6	isopropanol	79	80	79.5 ± 0.7
7	water	9	10	9.5 ± 0.7

**Table 2:** Estimated HSP of cutin based on swelling data from 5 and 6 solvents.

Values from 5 Solvents (Water and Isopropanol Have Been Excluded)			
	δ_d_, MPa^1/2^	δ_p_, MPa^1/2^	δ_hb_, MPa^1/2^
**1st repetition**	19.2	5.8	2.5
**2nd repetition**	20.1	5.0	2.5
**Average values**	**19.7**	**5.4**	**2.5**
values from 6 solvents (water has been excluded)			
	**δ_d_, MPa^1/2^**	**δ_p_, MPa^1/2^**	**δ_hb_, MPa^1/2^**
**1st repetition**	26.3	2.5	10.2
**2nd repetition**	32.2	2.5	9.4
**Average values**	**29.2**	**2.5**	**9.8**

**Table 3:** Average HSP and R_a_ distances of cutin and of six different groups of food components.

Group	δ_d_, MPa^1/2^	δ_p_, MPa^1/2^	δ_hb_, MPa^1/2^	R_a_, MPa^1/2^
carbohydrates	17.0 ± 1.4	15.3 ± 2.8	21.6 ± 1.6	22.4 ± 2.2
fats	16.3 ± 0.3	3.9 ± 0.9	6.7 ± 2.1	8.3 ± 1.1
amino acids	18.0 ± 1.0	9.4 ± 3.3	16.2 ± 3.4	15.0 ± 3.7
vitamins	17.1 ± 0.6	2.4 ± 1.8	3.6 ± 1.1	6.4 ± 0.7
polar essential oil components	17.6 ± 0.9	3.7 ± 0.5	7.9 ± 1.8	7.3 ± 1.1
non-polar essential oil components	16.8 ± 0.4	1.6 ± 0.6	2.9 ± 0.9	6.9 ± 0.8

**Table 4:** Light absorbance of sunflower oil which was in contact with cutin for 6-month period at room temperature. Sunflower oil which was not in contact with cutin was used as the blank sample for the UV-VIS measurement.

	**Wavelength, nm**		
	400	550	700
**Absorbance**	0.055	0.015	0.011

## Data Availability

The original contributions presented in this study are included in the article/Supplementary Materials. Further inquiries can be directed to the corresponding author.

## Supplementary Materials

The following supporting information can be downloaded at https://www.mdpi.com/article/10.3390/polym17212961/s1, Figure S1: Polymerization reaction of cutin; Table S1: HSP and Ra distances of cutin and various food components of six different groups.
