# Supplementary material for: Predicting Packaging Material–Food Interactions and the Respective Migration and Permeation Based on Hansen Solubility Parameters—A Case Study of Bio-Based Polyester Cutin
Source: Polymers (Basel). 2025 Nov 6;17(21):2961. doi: 10.3390/polym17212961 (PMC12608475; doi:10.3390/polym17212961)
Supplement: Supplementary file 1 [file polymers-17-02961-s001.zip › polymers-3942479-supplementary.pdf]

Supplementary Materials

Predicting packaging material-food interactions and the respective migration and permeation based on Hansen Solubility Parameters. A case study of the biobased polyester cutin

Costas Tsioptsias [1,*], Athanasios Goulas [1], Maria Tsini [1], Athanasia Zoglopiti [1], Anna Marinopoulou [1] and Vassilis Karageorgiou [1]

[1] Department of Food Science and Technology, International Hellenic University, 57400, Sindos, Greece; ktsiopts@ihu.gr (C.T.), agoulas@ihu.gr (A.G.), tsinimar@gmail.com (M.T.), amarinop@ihu.gr (A.M.), vkarageorgiou@ihu.gr (V.K.)

* Correspondence: ktsiopts@ihu.gr (C.T.)

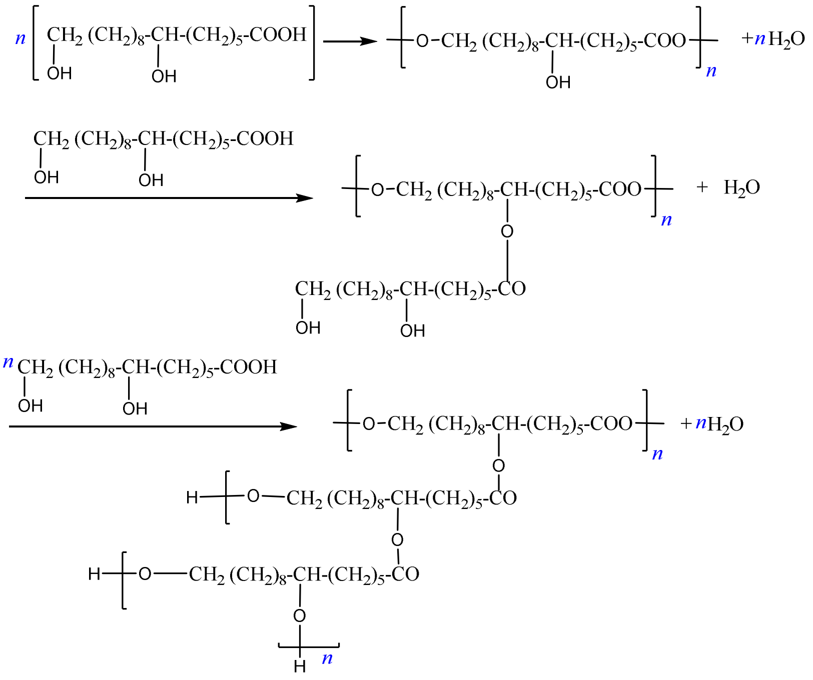

Figure S1. Polymerization reaction of cutin

Table S1. HSP and Ra distances of cutin and various food components from six different groups

	δ_d. MPa$^{1/2}$	δ_p. MPa$^{1/2}$	δ_{hb}. MPa$^{1/2}$	R_a. MPa$^{1/2}$
**carbohydrates**				
Lactose	15.6	16.9	20.4	22.8
Dextrose	17.9	13.7	23.6	22.9
Sucrose	15.9	18.4	21.9	24.5
Dextro-Xylose	18.5	12.3	20.3	19.2
**average**	**17.0**	**15.3**	**21.6**	**22.4**
**standard deviation**	**1.4**	**2.8**	**1.6**	**2.2**
**fats**				
Glyceryl Monostearate	16.1	4.5	9.8	10.2
Glyceryl Monooleate	16.2	4.6	9.4	9.8
Glyceryl Trioleate	16.0	3.8	3.2	7.5
Glyceryl Tripropanoate	16.4	5.6	7.2	8.0
Stearic Acid	16.3	3.3	5.5	7.6
Hexadecanoic Acid (palmitic acid)	16.3	3.4	6.0	7.8
Oleic Acid	16.0	2.8	6.2	8.6
Linoleic Acid	16.8	3.3	6.2	7.1
**average**	**16.3**	**3.9**	**6.7**	**8.3**
**standard deviation**	**0.3**	**0.9**	**2.1**	**1.1**
**amino acids**				
d-p-Hydroxyphenylglycine	20.0	9.9	19.5	17.6
Glycine	18.0	9.9	19.5	17.9
Phenylglycine	18.6	6.1	12.6	10.3
n-Leucylglycine	17.6	12.6	14.8	14.8
l-Proline	17.7	7.8	13.2	11.6
Alanine	17.1	7.9	16.9	15.5
l-Cysteine	18.4	9.3	17.3	15.5
Laevo-Histidine	19.4	12.3	16.7	15.8
Laevo-Arginine	17.7	14.7	15.5	16.4

l-Asparagine	18.5	16.3	21.5	22.0
Lysine	17.2	7.1	14.2	12.8
Laevo-Aspartic Acid	18.1	10.1	21.6	19.9
Laevo-Glutamine	18.2	15.0	19.6	19.8
l-Phenylalanine	18.3	5.7	11.7	9.6
Methionine	18.1	7.3	13.2	11.3
l-Tryptophan	19.7	8.2	12.8	10.7
l-Tyrosine	17.5	6.9	17.2	15.4
Threonine	17.3	9.6	21.0	19.5
Isoleucine	16.3	5.1	11.7	11.4
Valine	16.3	6.0	13.1	12.6
**average**	**18.0**	**9.4**	**16.2**	**15.0**
**standard deviation**	**1.0**	**3.3**	**3.4**	**3.7**
**vitamins**				
Vitamin A Palmitate	16.8	0.9	2.3	7.3
Vitamin D2	17.7	1.8	4.3	5.6
Vitamin K1	16.3	5.1	3.1	6.7
Cholecalciferol Vitamin D3	17.4	1.8	4.6	6.1
**average**	**17.1**	**2.4**	**3.6**	**6.4**
**standard deviation**	**0.6**	**1.8**	**1.1**	**0.7**
**polar essential oil components**				
Thymol	19.0	4.5	10.8	8.4
Menthol	16.5	3.6	7.6	8.3
Terpineol	17.1	3.6	7.6	7.4
Santalol	17.4	3.0	5.7	6.0
2-Methyl-5-Isopropylphenol (carvacrol)	17.8	3.9	7.6	6.5
**average**	**17.6**	**3.7**	**7.9**	**7.3**
**standard deviation**	**0.9**	**0.5**	**1.8**	**1.1**
**non-polar essential oil components**				
p-Cymene	17.3	2.3	2.4	5.6

β-Pinene	16.3	1.2	1.9	7.9
γ-Terpinene	16.7	1.8	3.7	7.0
d-Limonene	17.2	1.8	4.3	6.4
l-Limonene	16.7	1.8	3.1	6.9
Caryophyllene	16.8	0.6	2.2	7.5
**average**	**16.8**	**1.6**	**2.9**	**6.9**
**standard deviation**	**0.4**	**0.6**	**0.9**	**0.8**
